# Fun, influence and competence—a mixed methods study of prerequisites for high school students’ participation in physical education

**DOI:** 10.1186/s12889-017-4154-6

**Published:** 2017-03-10

**Authors:** Eirik Abildsnes, Gudrun Rohde, Sveinung Berntsen, Tonje H. Stea

**Affiliations:** 1 Department of Global Public Health and Primary Care, University of Bergen, Bergen, Norway; 2 Department of Health and Nursing Sciences, University of Agder, Kristiansand, Norway; 3 Department of Public Health, Sport and Nutrition, University of Agder, Kristiansand, Norway

**Keywords:** Adolescents, Healthy lifestyle, Focus groups, Physical activity, Health promotion

## Abstract

**Background:**

Many adolescents do not reach the recommended levels of physical activity (PA), and students attending vocational studies are less committed to take part in physical education (PE) than other students. The purpose of the present study was twofold: 1) to examine differences in physical activity, diet, smoking habits, sleep and screen time among Norwegian vocational high school students who selected either a PE model focusing on PA skills, technique and improvement of physical performance (“Sports enjoyment”) or more on health, play and having fun when participating in PE lessons (“Motion enjoyment”), and 2) to explore the students’ experiences with PE programs.

**Methods:**

In this mixed methods study 181 out of 220 invited students (82%) comprising 141 (78%) girls and 40 (22%) boys attending vocational studies of Restaurant and Food Processing (24%), Design, Arts and Crafts (27%) or Healthcare, Childhood and Youth Development (49%) were recruited for participation in the new PE program. PA level, sedentary time and sleep were objectively recorded using the SenseWear Armband Mini. A self-report questionnaire was used to assess dietary habits, smoking and snuffing habits, use of alcohol, screen use and active transportation. Four focus group interviews with 23 students (12 boys) were conducted to explore how the students experienced the new PE program.

**Results:**

Students attending “Motion enjoyment” accrued less steps/day compared to the “Sports enjoyment” group (6661 (5514, 7808) vs.9167 (7945, 10390) steps/day) and reported higher screen use (mean, 3.1; 95% CI, 2.8, 3.5) vs. 2.4 (2.0, 2.9) hours/day). Compared to those attending “Sports enjoyment”, a higher number of students attending “Motion enjoyment” reported an irregular meal pattern (adjusted odds ratio, 5.40; 95% confidence interval (CI), 2.28, 12.78), and being a current smoker (12.22 (1.62, 107.95)). The students participating in the focus group interviews emphasized the importance of having competent and engaging teachers, being able to influence the content of the PE program themselves, and that PE classes should include a variety of fun activities.

**Conclusion:**

Students selecting “Motion enjoyment” accrued less steps/day and reported overall more unhealthy lifestyle habits, including higher screen time, a more irregular meal pattern and a higher number were current smokers, compared to those selecting “Sports enjoyment”. Program evaluation revealed that both groups of students valued competent PE teachers and having influence on the content of the PE program.

## Background

Globally, 80% of adolescents do not reach the recommended levels of physical activity (PA) [1]. In Norway 87% of girls and 96% of boys participate in moderate intensity PA for at least 60 min a day at the age of 6 years, but at the age of 15 years only 43% of girls and 58% of boys reach this recommended level of PA [2]. Regular PA has beneficial short-term effects on physical, mental and social aspects of health among adolescents, and may improve self-esteem [3] and reduce symptoms of anxiety and depression [4]. Participation in PA has also been positively associated with academic performance in children and adolescents [5, 6]. Results from tracking of PA level indicate that a physically active lifestyle develops in early childhood and that the stability of PA is moderate or high from youth to adulthood [7]. Furthermore, strong evidence show long-term effects of PA in adulthood on rates of major non-communicable diseases, life expectancy, and the cost of health care spending [8, 9].

Schools may serve as an important arena for achieving public health goals in conjunction with their educational commitments [10]. Physical education in schools is universally applicable, and a systematic review has concluded that school-based programs might increase the students’ level of moderate-to-vigorous intensity physical activity (MVPA) [11]. Another systematic review of follow-up studies concluded that studies based on a theoretical framework and lasting longer than 1 year, were effective in producing sustained impact on PA habits and most likely on fundamental movement skills [12]. Results from another study have indicated a positive impact of early required physical education (PE) upon adult PA [13].

Self-determination Theory (SDT) identifies autonomy, perceived competence and relatedness as fundamental psychological needs [14]. Students enjoy participation in PE when they have a choice of activities, feel competent, in control and supported by their peers and teachers [15]. Determinants of PA level in a population includes individual factors such as age, sex, health status, self-efficacy, and previous PA as well as physical and environmental factors like economic conditions, societal norms, urbanisation and industrialisation [16]. Studies have suggested that the following important factors should be included as essential parts of PA and PE programs; involvement of competent PE teachers, support from family and community stakeholders, as well as considerations about how social, behavioural, demographical, biological, environmental, psychological and cognitive factors influence PA [17, 18]. Furthermore, adolescents should be encouraged to take part in program development, selecting activities that generate fun, enjoyment and interest [19, 20]. PE programs based on these principles may support intrinsic motivation and adherence to PA [21, 22]. It is also suggested that knowledge about the target population’s background and preferences should be included in intervention studies of PA among adolescents [23].

The intention of PE programs in Norwegian high schools is to inspire students to experience enjoyment and lifelong adherence to participation in PA, and develop a positive perception of body, self and identity [24]. To reach this goal, design of PE programs should intend to facilitate adherence to PA in general. A report from the World Health Organization (WHO) describes that adults with vocational education are less physically active than adults with college or university background in developed countries [25]. Thus, interventions intending to increase PA among adolescents should especially focus on students attending vocational studies. Even though attending PE is mandatory in Norwegian high schools, we in a previously published paper from the present study found that vocational students attend PE less frequently; have lower grades in PE and more frequently drop out of school than other high-school students [26, 27].

Few previously published studies have evaluated students’ experiences of participation in PE programs and examined differences in objectively measured PA and between students choosing different PE models. In the present study, we compared students choosing a PE model based on personal preferences, focusing on PA skills, technique and improvement of physical performance (“Sports enjoyment”) or more on health, play and having fun (“Motion enjoyment”), respectively. Specifically, we intended to 1) examine differences in MVPA, sedentary time, steps, sleep hours, screen time, diet, tobacco and alcohol use among students who selected the PE models “Sports enjoyment” and “Motion enjoyment”, and 2) explore how the students experienced PA, PE and the new PE program.

## Methods

In August 2013, two high schools in southern Norway introduced a new PE program to students attending their first year of vocational studies and invited the researchers to evaluate the new PE program. The students chose to participate in one out of two alternative PE models; 1) “Sports enjoyment” focusing on PA skills, in-depth knowledge of different sports, technique and improvement of physical performance, and 2) “Motion enjoyment” focusing less on technique and physical performance, but more on health benefits, achieving new experiences with PE, playing and having fun with sufficient intensity when participating in PE lessons. In the latter model, three groups of students attended PE class simultaneously, and the students could choose among three different activities. Three well-qualified PE teachers guided the activities. The students had to attend the chosen activity for the following 3 weeks, and then select another activity.

All students received the following information before choosing PE model: “If you choose “Motion enjoyment” PE class will contain activities that promote the joy of being physically active. If you choose “Sports enjoyment” PE class will contain more activities that develop your skills in different sports”. The students attended PE class twice a week and had a total of 56 h of PE a year.

We used a mixed method study design. Data collection methods included both quantitative and qualitative measures to examine selected lifestyle habits of the participants and evaluate the PE program described above. A self-reported questionnaire was used to collect data and investigate differences in PA, dietary habits and tobacco use of the students selecting different PE models, and focus group interviews explored the students’ adherence to PA and how they experienced the new PE program.

The mixed method analysis strategy represents a parallel mixed data analysis, with separate quantitative and qualitative strands implemented to answer related aspects of the research questions regarding the same phenomenon [28]. Inferences of each strand are integrated into meta-inferences at the end of the study, and presented in the discussion section.

The target group was students attending their first year of vocational studies at two high schools in the south of Norway, where PE teachers had experienced low student commitment in participating in PE [29]. During their first week at high school, the students received information about the PE program at meetings, via the intranet and by written information. After 6 weeks, the students were required to decide which PE model they wanted to join. During their first year in high school the students attending “Motion enjoyment” could choose between two or three different activities, lasting 3 weeks each. They were introduced to activities like climbing, swimming, yoga, orienteering and different ballgames. Some activities like dancing, outdoors and different net games were mandatory for both groups. The “Sports enjoyment” groups were introduced to different sports during the year, and focused on techniques and performance. Students who had an injury or other health complaints were taken care of by one of the teachers, finding some activity the student could manage. The students received evaluation with a 6-point grading scale with 6 as the best result. Evaluation was based on the students’ efforts and achieved competence in the three main subject areas of the PE curriculum. These areas included exercise and lifestyle, outdoor life and sports activities.

In August 2013, 181 out of 220 invited students in 9 classes comprising 141 (78%) girls and 40 (22%) boys attending vocational studies of Restaurant and Food Processing (24%), Design, Arts and Crafts (27%) or Healthcare, Childhood and Youth Development (49%) were recruited for participation in the new PE program. In September 2013 all students were invited to fill in a questionnaire and no students were excluded. A total of 101 (24 boys) of the students (17 years old (SD 2.4)) agreed to wear a PA monitor (SenseWear™ Armband Mini, BodyMedia Inc., Pittsburgh, Pennsylvania, USA) for seven consecutive days in October 2013–January 2014. Furthermore, 23 of the students participating in the quantitative baseline study accepted to participate in a focus group study at the end of the intervention period in March–April 2014. Twelve of these students (6 boys) attended “Motion enjoyment” and 11 students (6 boys) attended “Sports enjoyment”. The mean age of the students was 17 years (SD 2.6).

### Quantitative instruments

#### Physical activity

The SenseWear Armband Mini (SWA) is a portable device that includes physiological parameters, including heat flux, skin temperature, galvanic skin response and movement (tri-axial accelerometer). SWA has been found valid for recordings of different aspects of physical activity as MVPA and sedentary time in a variety of populations including children and adolescents [30–33]. The participants were instructed to wear the SWA for seven consecutive days. They were instructed on how to wear the SWA and that it should be worn at all times except when taking a bath or shower. The SWA was worn on the left arm over the triceps branchii muscle at the midpoint between the acromion and olecranon processes. Data was downloaded and analysed with the manufacturer’s software (SenseWear™ Professional Research Software Version 8.1, BodyMedia Inc.). The summed value of 1-min epochs was used to estimate metabolic equivalents (METs) (1 MET = 3.5 ml O_2_ · kg^−1^ · min^−1^). Cut points defining MVPA were ≥3 METs, cut points defining light PA were 1.6–2.9 METs and accumulated minutes were included. Sedentary time was defined as METs ≤1.5. A day of recording was valid if the participant wore the SWA for at least 19.2 h, i.e. 80% of a 24-h sampling period [34]. In the analysis we included only recorded data from adolescents with ≥1 valid day of SWA wear time.

#### The questionnaire

The questionnaire was designed in the web-based online platform SurveyXact^TM^ (Rambøll, Management Consulting, Oslo, Norway). The students used approximately 20–30 min to complete the questionnaire, with at least one member of the project group present to answer upcoming questions. To access the questionnaire, the participants had to open a webpage and enter the provided identification key.

The questions used in the present study has, in a previously published method study among 143 adolescents, aged 15–17 years, demonstrated test-retest reliability with an intraclass correlation coefficient (ICC) ranging from 0.68 to 0.99 [6].

The parental educational level was assessed with the question: “*What level of education do your parents have?*” The question had four response alternatives: (i) elementary school, (ii) high school, (iii) college or university (≤3 years) and (iv) college or university (>3 years). These response alternatives were then dichotomized into lower and higher education levels (lower = no college or university education; higher = having attended college or university).

Meal frequency was assessed by the questions such as: “How often do you have breakfast each week?” The same was asked for lunch, dinner and evening meals. The items had five different response alternatives ranging from never to daily, which were dichotomized into having meals fewer than seven times a week (an irregular meal pattern) and having meals every day (a regular meal pattern). These dichotomous variables were then combined to create a summary variable referred to as “irregular all four meals”, i.e. those skipping one of the main meals at least once a week versus those eating all meals every day.

Diet and beverage intake was assessed by asking: *“How often do you eat/drink…?”* All items had eight different response alternatives, ranging from never to more than once a day, and the response alternatives were further dichotomized into having high or low intake of the selected food items and drinks. Having soft drinks, sweets and candy and a salty snack ≥ 4 times a week, respectively, was categorized as high intake. Having fruits, berries and vegetables less than once a day, was categorized as low intake.

Alcohol use was assessed by asking: *“Have you ever consumed beer, wine or spirits?”*

Smoking and snuffing habits were assessed by the question: “*Do you smoke/use snuff?”* The response alternatives were: *“Have never smoked/snuffed; have tried smoke/snuff, but not anymore, have smoked/snuffed regularly, but not anymore; smoking/snuffing, but not regularly* and *smoking/snuffing regularly and about__ cigarettes/day.”* For the statistical analysis, those who reported smoking or snuffing occasionally or daily were classified as being a smoker/snuffer.

Television viewing and computer use was assessed as follows: *“Excluding school hours on a regular weekday, how many hours do you watch TV or use PC/games?”.* Response alternatives were: “I don’t watch TV or do gaming activity on a regular weekday, less than an hour a day, 1 h a day, 2 h a day, 3 h a day or 5 h or more a day” and presented as a continuous variable.

Information about active commuting was enquired about as follows: “*How do you usually commute to/from school?”* The response alternatives were: *“Walking, cycling, bus, car, MC/scooter, other alternatives (open alternatives).”* This variable was dichotomized into active commuting, which represented walking or cycling and non-active commuting.

#### Statistical Analysis

All statistical analyses were performed using SPSS version 22.0 (SPSS Inc., Chicago, IL). Statistical significance level was set to 5%. Differences in continuous outcome variables (Table 1) between the two groups were assessed by analysis of covariance adjusting for age, parental education and mean valid days with recordings the SWA was worn (for the variables MVPA, steps, sedentary time and sleep hours). Adjusted means with 95% CI are presented. The underlying assumptions of the analysis of covariance were assessed using Jackknife Residuals and Cook’s d. The results are stratified on choice of PE model, presented as a percentage of participants reporting an irregular meal pattern, low intake of healthy food items and high intake of unhealthy food items and beverages in addition to being a current smoker or snuffer. Multiple logistic regressions were used to explore whether attending the PE model, “Motion enjoyment” was significantly associated with the health risk behaviors mentioned above, compared to attending “Sports enjoyment”.

### Qualitative data

We arranged 4 focus groups with 23 students (12 boys) attending “Sports enjoyment” and “Motion enjoyment”, respectively in March and April 2014, when the students had participated in the new PE program for approximately 6 months. We audiotaped and transcribed the audiotapes verbatim. In analyses we used Systematic Text Condensation and an editing analysis style [35, 36]. Bracketing preconceptions, two researchers independently read the material searching for an overall impression and established preliminary subthemes. We then examined the text for units of meaning representing information about students’ experiences with PA and PE and the new PE program. In an iterative process we coded and grouped these units, contrasted and abstracted the content in each group, and finally discussed and summarized the content of each group into generalized descriptions. To support analysis we used field notes and created mind maps, and discussed the analysis at each step to reach agreement. Quotations were used to illustrate and support findings. More information about the qualitative methods used is available from the interview guide and a COREQ checklist [37] (Appendices 1 and 2).

## Results

### Quantitative data

Students who chose to enroll in “Sports enjoyment” accrued more steps/day compared to the “Motion enjoyment” group (9167 (7945, 10390) vs. 6661 (5514, 7808) steps/day) (Table 1). Neither MVPA, light PA, sedentary time, nor sleep hours were significantly different between the two groups of students (Table 1). The mean grades were 3.6 (3.1–4.2) and 2.8 (2.5–3.1) for students selecting “Sports enjoyment” and “Motion enjoyment”, respectively.

All results from the questionnaire were adjusted for age, gender and parental education. Students attending “Motion enjoyment” reported higher TV/PC use compared to “Sports enjoyment” (mean ± 95% CI; 3.1 (2.8, 3.5) vs. 2.4 (2.0, 2.9) hours/day) (Table 1). 82.6% (95%CI; 75.8, 89.5) attending “Motion enjoyment” and 77.1% (64.8, 89.4) attending “Sports enjoyment” reported no active commuting to school (Table 2).

**Table 1:** Adjusted ^a^ hours in daily moderate to vigorous intensity physical activity, steps, sleep and screen hours (TV/PC) presented by motion enjoyment and sport enjoyment. Data are given as adjusted means with 95% confidence intervals (95% CI) in parentheses

	Motion enjoyment Mean (95% CI)	Sport enjoyment Mean (95% CI)	*P*-value*
Moderate-to-vigorous intensity PA (hours · day^-1^)	1.84 (1.42, 2.27)	2.21 (1.76, 2.67)	0.138
Steps per day	6661 (5514, 7808)	9167 (7945, 10390)	**<0.001**
Light PA (hours · day^-1^)	4.7 (3.6, 5.9)	4.6 (3.4, 5.9)	0.893
Sedentary time (hours · day^-1^) ^b^	11.1 (9.8, 12.4)	10.7 (9.3, 12.1)	0.572
Sleep (hours · day^-1^)	7.1 (6.7, 7.5)	6.8 (6.4, 7.2)	0.228
TV/PC (hours/day) ^c^	3.1 (2.8, 3.5)	2.4 (2.0, 2.9)	**0.006**

*Abbreviations*: *PA* Physical Activity

^*^ *P*-values for differences between groups, significant *p*-values given in bold

^a^ Adjusted for age, sex and parental education and days with recordings

^b^ Sedentary time, 06:00–23:59

^c^ Data from questionnaire

**Table 2:** Prevalence and adjusted odds ratio (AOR) of selected health-risk behaviors by participation in different models of physical education. Significant differences between groups is given in bold

	“Motion enjoyment”		“Sport enjoyment”		*AOR* ^*a*^	*95% CI*
	*%*	*95% CI*	*%*	*95% CI*		
Irregular breakfast	70.3	62.0–78.5	55.3	40.6–70.1	1.73	0.82–3.63
Irregular lunch	68.6	60.2–77.0	59.6	45.0–74.1	1.51	0.72–3.17
Irregular dinner	40.5	31.6–49.4	29.8	16.2–43.4	1.87	0.86–4.09
Irregular evening meal	75.2	67.4–83.0	63.8	49.6–78.1	1.97	0.91–4.28
Irregular meal pattern (<4 meals per day)	90.9	85.7–96.1	80.9	69.2–92.5	**5.40**	**2.28–12.78**
Low fruits and berries (<7 times a week)	75.2	67.4–83.0	66.7	52.8–80.5	1.65	0.75–3.64
Low vegetables (<7 times a week)	76.9	69.2–84.5	64.6	50.6–78.6	2.15	0.98–4.70
High regular soft drinks (≥4 times a week)	32.2	23.8–40.7	27.1	14.0–40.1	1.42	0.63–3.20
High sweet and candy (≥4 times a week)	26.5	18.5–34.4	18.8	7.3–30.2	1.16	0.48–2.81
High salty snack (≥4 times a week)	14.9	8.4–21.3	25.0	12.3–37.7	0.47	0.19–1.14
Have been drinking alcohol	64.5	55.8–73.1	63.8	49.6–78.1	1.30	0.60–2.80
Current smoker^b^	19.0	11.9–26.1	2.1	0.0–6.4	**13.22**	**1.62–107.95**
Current snuffer^b^	22.3	14.8–29.8	14.9	4.3–25.5	1.25	0.48–3.28
No active commuting to school	82.6	75.8–89.5	77.1	64.8–89.4	1.68	0.67–4.21

^a^ Adjusted for age, sex and parental education

^b^Self-reported smoking/snuffing sometimes or daily

Although the results showed no difference in consumption of single meals, attending “Motion enjoyment” was associated with irregular meal pattern (skip one or more of the main four meals during a day) (adjusted OR 5.40; 95% CI, 2.28, 12.78) (Table 2). There were no differences in intake of fruit and berries, vegetables, soft drink, candy and salty snack, nor alcohol consumption debut between participation in the different PE models (Table 2). On the other hand, attending “Motion enjoyment” was associated with being a current smoker (13.22; 1.62, 107.95), but not snuffer (Table 2).

### Qualitative data

All students described their previous and current experiences with PA and PE, and how they perceived their peers’ attitudes towards taking part in PE class. They also expressed their preferences for PE. The qualitative results represent opinions of the students who participated in the focus groups.

#### Previous PA and PE experiences

The students attending both PE models reported diverging previous experiences with leisure-time PA and PE in junior high school, but the students attending “Sports enjoyment” more often reported positive engagement in leisure time PA. These previous experiences with PE and PA seemed to influence the students’ attitudes towards PE. Some students attending both models reported low attendance to PE in their junior high school class, and that it had been easy to shirk PE class by faking injuries or sickness, or by deliberately forgetting fitness gear. Others reported high level of attendance and positive experiences of participating in PE. These students, attending both PE models, also had experienced devoted and inspiring PE teachers, and a variety of activities in PE class.

*The students in my class [in junior high school] were eager to exercise, so everyone participated. It was fun. Some students participated less than others, but the PE teacher was aiming to get all students to be physically active (boy, “Motion enjoyment”)*

Other students described their PE teachers in junior high school as being lazy and not organizing stimulating PE activities. All students, however, reported limited expectations and experiences of learning outcome from PE in junior high school, and did not consider PE as a subject intending to increase competence of health and physical literacy.

The girls attending “Sports enjoyment” told about groups of other girls who skipped PE class because they did not want to expose an obese body, mess up their make-up or have to shower after class. The boys attending both PE models explained their own non-attendance in high school by injuries. The boys attending “Motion enjoyment” also reported missing the school bus, having back pain or having overslept when explaining non-attendance.

The students also told about their previous and present leisure time PA engagement. Some of the girls attending “motion enjoyment” described that they preferred to exercise at home or alone, doing yoga or running on a treadmill, and avoided visiting gyms or to participate in organized sports. These students often reported to take part in PA and PE to stay healthy. The boys attending this PE model most of all expected fun and enjoyment in PE class, but some of them wished to increase strength. Girls and boys attending “Sports enjoyment” more regularly participated in organized sports activities, and expressed ambitions of increasing physical fitness and strength. These students more often enjoyed visiting a gym,. Due to time constraints and part-time jobs, sports injuries or other health complaints, students attending both PE models had stopped attending organized sport activities when starting high school. Others had dropped out because they experienced too high ambitions in organized sport, even if they enjoyed being physically active and attending PE classes.

*I actually do nothing. I have tried many different activities, but never committed to a single sport activity or a gym (girl, “Sports enjoyment”)*

#### New PE experiences

Overall, the students attending both PE models reported positive experiences with the new PE program. They appreciated being able to choose “Motion enjoyment” or “Sports enjoyment”, being offered a variety of PE activities, and that PE teachers took into account that some students did not fancy traditional PE. All focus group participants reported a higher intensity in PE classes than they were used to in junior high school, and that most of their peers participated in PE classes. Students representing both PE programs appreciated that grading was mainly based on efforts rather than physical achievements, but some of them asked for tests to monitor improvement in physical fitness.

*If I just try to do my best now, I get better grades than I did in junior high school. Then I try even more. It is more fun to do my very best now (boy, “Motion enjoyment”)*

The girls attending “Motion enjoyment” appreciated being introduced to yoga. They described that yoga made them use several groups of muscles, and made it easier to relax and to cope with stress.

*If you have had a bad day or had a lot of stress, it [yoga] can be delicious (girl, “Motion enjoyment”)*

Boys attending “Motion enjoyment” preferred other activities than yoga, but described that they experienced increased self-efficacy and enjoyment, and appreciated the variety of activities in PE class. The boys attending “Sports enjoyment” reported high intensity and engagement, a focus on improving techniques and succeeding to play as a team. All focus group participants reported that theoretical aspects of physical function and health was addressed in other study subjects, but reported some focus on diet and physical activity in PE class. A few of the students reported increased leisure time PA and physical fitness due to individual advice from their PE teacher. The girls attending “Sports enjoyment” enjoyed taking part in PE class, and appreciated a variety of activities.

*More people take part in PE class. It is positive that more girls participate [in PE class]. It used to be opposite. Earlier, more boys attended. Now, it is more girls (girl, “Sports enjoyment”)*

The students also reported some negative experiences related to the new PE program. In the first period after implementation of the new PE models, the organization of PE classes was somewhat chaotic; the students experienced random decisions of individual grading and registration of attendance. Later on, the students reported that the organization of PE classes improved. More girls than boys attended to “Motion enjoyment”. The boys attending this model missed activities intending to increase muscle strength, and in their opinion, this PE model was more tailored to girls’ interests than boys’.

*To be honest, I miss an option of doing some real exercise. Even if it is “Motion enjoyment” (boy, “Motion enjoyment”)*

Even if the PE program intended to tailor activities to fit the students’ needs and interests, the students reported that they did not perceive that the program fully elicited the potential of each individual.

#### Wishes for PE teaching

All students recognized the PE teacher’s role to be of crucial importance. They characterized the teacher as an important role model, and the teacher being in a power position in the student-teacher relationship. Teachers with professional skills and commitment, who recognized and followed up students individually and offered a variety of activities in PE class, were highly appreciated. The students also stated that they had a responsibility to engage themselves, to experience fun and enjoyment in PE class.

*The teachers are clever and know what they are doing. If I needed help, then they help me. We have fun activities that I like, like climbing and swimming. So it is very nice (boy, “Motion enjoyment”)*

Students participating in both PE models appreciated the variation of PE activities included in PE classes, and also the opportunity to choose between the two models of PE. Although the students had different preferences with respect to what kind of activities they liked, they underscored a wish to participate in developing the program content. All focus group participants suggested that the PE classes should provide theory as well as practical information about diet and PA, and also a focus on injury prevention. They appreciated that students with injuries or other health complaints were offered individually tailored activities when they were unable to participate in regular PE classes.

*Maybe the students could give some input to what we shall do in PE class. Of course the teacher shall decide, but it would be nice if the students had some influence. In the end, they are the ones who shall take part in PE class (girl, “Motion enjoyment”)*

## Discussion

In the present study, we compared students choosing a PE model based on personal preferences, focusing on PA skills, technique and improvement of physical performance (“Sports enjoyment”) or more on health, play and having fun (“Motion enjoyment”), respectively. Specifically, we intended to 1) examine differences in MVPA, sedentary time, steps, sleep hours, screen time, diet, tobacco and alcohol use among students who selected the PE models “Sports enjoyment” and “Motion enjoyment”, and 2) explore how the students experienced PA, PE and the new PE program.

A majority of students in both groups met the recommendations of MVPA. However, students attending “Sports enjoyment” accrued more steps/day than those attending “Motion enjoyment”. These results were supplemented by findings from the focus group interviews, which showed that students attending “Sports enjoyment” were more often engaged in sport clubs and competitions, whereas those attending “Motion enjoyment” preferred to exercise at home or alone and did not visit gyms or participate in organized sport. All students did not meet the recommended level of PA, and results from the focus groups also revealed decreased participation in PA with age among all students. These findings are in line with the results from a cohort study, which showed a decreased level of MVPA between 5 and 19 years of age [38]. In addition, the latter mentioned study identified four different MVPA patterns: 1) consistently inactive (14.9%), 2) consistently active (18.1%), 3) decreasing moderate intensity PA (52.9%), and 4) substantially decreasing vigorous intensity PA (14.1%). In the present study the students who participated in the focus groups described various reasons for stopping exercising with age, including time constraints as well as injuries and experiencing too high ambitions in organized sports.

Selecting “Motion enjoyment” was associated with a number of unhealthy lifestyle habits, including irregular consumption of main meals, smoking cigarettes and an increased number of cigarettes per day and an increased screen time. In line with the results from the present from the present study, a comprehensive review has confirmed that diet, PA and sedentary behaviours tend to cluster in both healthy and unhealthy behaviours, and that cluster patterns varied according to age, gender and socio-economic status [39]. Lifestyle habits adopted in childhood and adolescence may have impact on health in adulthood [7]. Thus the findings in the present study may perhaps reflect individual preferences, previous experiences with PA and social learning.

Previously published results based on findings reported by the same target group have shown a regular meal pattern, a high intake of healthy food items and being physically active were associated with increased odds of high academic achievement [6]. Having an irregular meal pattern is common among high school students, especially among vocational students [40]. Analyses based on both questionnaire data and interview data in the present study also indicated that the students choosing “Motion enjoyment” could benefit from an increased focus on different health behaviors, and despite low expectations of increased knowledge about healthy lifestyle habits, students participating in focus group interviews expressed a positive attitude toward learning more about healthy dietary habits, in addition to injury prevention and how to increase muscle strength in PE classes. Thus a potential exists for including an increased focus on theory and practical knowledge to increase physical literacy and health literacy in PE that may have an impact on future health risks [10].

In order to motivate participation in PE class, students participating in both PE models emphasized the importance of having a competent and engaging teacher, and including a varied selection of fun activities in PE class. A recently published systematic review confirmed that the content and design of PE programs may influence adherence to PE class and learning outcomes [18]. Furthermore, students who participated in focus group interviews expressed various experiences with leisure time PA and PE programs in junior high school. These experiences may also have influenced their choice of PE model in high school, and possibly their commitment to PA in general. A review study by Lai et al indicate that PE programs in schools have a potential to improve movement skills, generate fun, positive experiences and commitment to PA [12].

In the present study, mean MVPA was 1.8 h a day in the “Motion enjoyment” group vs. 2.2 h a day in the “Sports enjoyment” group. Thus many students in both groups met the recommendations of PA [2]. In a longitudinal study of PA habits among Norwegian adolescents, 85% of boys reported being physically active at least two–three times per week at age 16 and 52% at age 19. Corresponding proportions for girls were 56 and 46%, respectively [41]. The students who attended the focus groups explained that they spent less leisure time on PA due to time constraints and part-time jobs when starting high school. Others experienced organized sport as being too ambitious, even if they enjoyed being physically active and attending PE classes. Healthy lifestyle habits and PA levels are associated with academic achievements among adolescents [6], and lifestyle habits adopted in childhood and adolescence have impact on health in adulthood [7]. Thus positive experience of PE in high school is valuable with respect to health as well as academic results.

The students who participated in the focus groups underscored the importance of a competent and supporting PE teacher, who was willing to let the students influence the development of the PE program. This is in line with the basic psychological needs described in SDT, and supports the findings from a study of Bagøien et al [42]. In their study of Norwegian high school students’ perceptions of PE, the importance of autonomy-supportive teachers in PE was positively associated with students’ psychological needs satisfaction in PE and positively related to autonomous motivation for PE participation. In a previous published paper from the present study, PE teachers stated that they wanted to give their students an influence on the content of the PE program and facilitate fun and engagement without compromising with the framework of the education program and curriculum [27].

Strengths of the present study included the use of objectively measured PA, the high response rate, and the mixed method approach, which provided rich data on lifestyle-related topics and commitment to PA and PE. In addition, a multidisciplinary team of researchers conducted the study in close cooperation with PE teachers in an everyday PE setting. Limitations include a small and selective study sample, a cross sectional design that did not allow us to compare effects of the two models, and lack of longitudinal data. Because of the cross sectional design, we are unable to determine whether differences between the two groups are due to the classes themselves, or could be explained by pre-existing differences between students who chose to enroll in one program over the other. The measured behaviors could also be confounded with the PE class students were attending. In addition, the majority of the students were girls, and all vocational study programs were not included. A small number of boys limit the possibility to analyze gender differences. Finally, the questionnaire and objective PA were not measured simultaneously, and the period of PA assessment was long due to limited access to PA monitors. Thus the internal and external validity of the study is limited.

The present has provided increased knowledge about important factors when planning future PE programs according to the preferences and expectations of students. In line with the present study, a study from van Sluijs and Kriemler [23] has confirmed that when designing PE programs, students’ background, preferences and physical activity experiences has to be kept in mind as a prerequisite for PE programs that generate fun and support motivation for enjoying regular PA. In the present study, students participating in the focus groups also suggested to include theory, by providing information concerning the relationship between lifestyle habits and physical and mental health. Thus it should be possible to take advantage of PE as an opportunity to inspire adolescents to adopt and maintain a healthy lifestyle. The probability of success may depend competent PE teachers who provide PE the students perceive as meaningful and fun, and to include the students’ preferences in program development.

## Conclusion

The results indicated that students selecting “Motion enjoyment” accrued less steps/day, reported a higher screen time, a more irregular meal pattern and a higher number were current smokers compared to students selecting “Sports enjoyment”. Program evaluation revealed that both groups of students highlighted the importance of having competent PE teachers, being able to influence on the content of the PE program and that PE classes should include a variation of fun activities.

## Appendix 1

### Interview guide

How are your previous experiences with physical education and physical activity?

- Enjoyment
- Participation
- Leisure-time physical activity
- Physical education in junior high school

How are your experiences with physical education in high school so far?

- Enjoyment
- Participation

What have you learned in physical education?

- Knowledge of health and physical activity?
- «Technical skills »?

Some students need tailoring to be able to participate. How do you think this works?

- What is wanted?
- What is possible to do?

How should the education be to make most students enjoying to participate?

- Motion enjoyment
- What do you thing about our PE model?

## Appendix 2

### Consolidated criteria for reporting qualitative studies (COREQ): 32-item checklist

Developed from:

Tong A, Sainsbury P, Craig J. Consolidated criteria for reporting qualitative research (COREQ): a 32-item checklist for interviews and focus groups. *International Journal for Quality in Health Care*. 2007. Volume 19, Number 6: pp. 349–357

**Table:**

No. Item	Guide questions/description	Response
Domain 1: Research team and reflexivity		
*Personal Characteristics*		
1. Interviewer/facilitator	Which author/s conducted the interview or focus group?	EA, and GR conducted the focus groups supported by an assistant (CSO)
2. Credentials	What were the researcher’s credentials? E.g. PhD, MD	EA: MD, PhD. GR: MSc, PhD. CSO: MSc
3. Occupation	What was their occupation at the time of the study?	EA: Public health officer. GR: Professor. CSO: Physiotherapist
4. Gender	Was the researcher male or female?	Female: GR, CSO. Male: EA
5. Experience and training	What experience or training did the researcher have?	The group of researchers had experience with qualitative and quantitative research methods based on several previous research projects.
*Relationship with participants*		
6. Relationship established	Was a relationship established prior to study commencement?	No
7. Participant knowledge of the interviewer	What did the participants know about the researchers? e.g. personal goals, reasons for doing the research	The participants knew that members of the research group were interested in adolescent health, and had signed an informed consent prior to participation
8. Interviewer characteristics	What characteristics were reported about the inter viewer/facilitator? e.g. Bias, assumptions, reasons and interests in the research topic	The interviewers represented different professions: Medicine (EA), physiotherapy (CSO) and nursing science (GR)
Domain 2: study design		
*Theoretical framework*		
9. Methodological orientation and Theory	What methodological orientation was stated to underpin the study? e.g. grounded theory, discourse analysis, ethnography, phenomenology, content analysis	Systematic Text Condensation represents a hermeneutic phenomenological approach. Self-determination theory was used as a theoretical framework
*Participant selection*		
10. Sampling	How were participants selected? e.g. purposive, convenience, consecutive, snowball	PE teachers invited a convenient sample of students participating in the study to take part in focus group interviews
11. Method of approach	How were participants approached? e.g. face-to-face, telephone, mail, email	The participants were invited face-to-face by their PE teacher an encouraged to participate in focus group interviews
12. Sample size	How many participants were in the study?	We arranged 4 focus groups:
		Six boys attending “Motion enjoyment”
		Six girls attending “Motion enjoyment”
		Five girls attending “Sports enjoyment”
		Six boys attending “Sports enjoyment”
13. Non-participation	How many people refused to participate or dropped out? Reasons?	Two boys attending “Sports enjoyment”, one girl attending “Sports enjoyment”, 2 girls attending “Motion enjoyment” and two boys attending “Motion enjoyment” did not show up
*Setting*		
14. Setting of data collection	Where was the data collected? e.g. home, clinic, workplace	The interviews took place in meeting rooms at the schools during school hours
15. Presence of non-participants	Was anyone else present besides the participants and researchers?	No, only the researcher or the research assistant
16. Description of sample	What are the important characteristics of the sample? e.g. demographic data, date	Male and female students attending to both PE programs participated
*Data collection*		
17. Interview guide	Were questions, prompts, guides provided by the authors? Was it pilot tested?	The interview guide is enclosed with the manuscript.
18. Repeat interviews	Were repeat inter views carried out? If yes, how many?	No
19. Audio/visual recording	Did the research use audio or visual recording to collect the data?	The interviews were audiotaped
20. Field notes	Were field notes made during and/or after the inter view or focus group?	Short field notes were made after the interviews
21. Duration	What was the duration of the inter views or focus group?	The duration of the focus group interviews were 45–90 min.
22. Data saturation	Was data saturation discussed?	Data saturation was discussed and considered sufficient to perform the analysis
23. Transcripts returned	Were transcripts returned to participants for comment and/or correction?	No
Domain 3: analysis and findings		
*Data analysis*		
24. Number of data coders	How many data coders coded the data?	Two researchers (EA and GR) coded the data
25. Description of the coding tree	Did authors provide a description of the coding tree?	The headlines and subtitles in the results presentation represent the final coding tree.
26. Derivation of themes	Were themes identified in advance or derived from the data?	Themes were derived from the data.
27. Software	What software, if applicable, was used to manage the data?	We used NVivo® for Mac version 10.2.1 to assist analysis.
28. Participant checking	Did participants provide feedback on the findings?	No
*Reporting*		
29. Quotations presented	Were participant quotations presented to illustrate the themes/findings? Was each quotation identified? e.g. participant number	Yes. Gender and PE program identified the participants
30. Data and findings consistent	Was there consistency between the data presented and the findings?	Yes
31. Clarity of major themes	Were major themes clearly presented in the findings?	Yes
32. Clarity of minor themes	Is there a description of diverse cases or discussion of minor themes?	Several diverse cases and minor themes are described in the results chapter

## Abbreviations

CI: Confidence interval
MVPA: Moderate-to-vigorous intensity physical activity
OR: Odds ratio
PA: Physical activity
PE: Physical education
SDT: Self-determination theory
WHO: World Health Organization
